# Reliable ECG monitoring during rest and exercise: a pilot comparative validation of a wearable single-lead band

**DOI:** 10.1093/ehjimp/qyag109

**Published:** 2026-06-15

**Authors:** Valentina Barletta, Matteo Bartalucci, Federico Starace, Andrea Ripoli, Riccardo Ceccarelli

**Affiliations:** UO Cardiologia 2, CardiacThoracic and Vascular Department, Azienda Ospedaliero Universitaria Pisana, Via Paradisa 2, Pisa 56100, Italy; Formula Medicine, Viareggio 55049 (LU), Italy; Unità di Sanità Pubblica, Dipartimento di Scienze Biomediche, Metaboliche e Neurali, Università di Modena e Reggio Emilia, Italy; Bioengineering Unit, Fondazione Toscana Gabriele Monasterio, Pisa 56124, Italy; Formula Medicine, Viareggio 55049 (LU), Italy

**Keywords:** electrocardiography, wearable electronic devices, Holter monitoring, exercise test, heart rate/RR interval

## Abstract

Wearable electrocardiogram (ECG) technologies have emerged as promising tools for continuous cardiac monitoring during physical activity. However, evidence regarding the accuracy of single-lead wearable ECG systems under dynamic exercise conditions remains limited. We evaluated the performance of a wearable chest-band ECG device by comparing simultaneous recordings with a conventional multi-lead exercise ECG system during rest and graded cycle ergometer exercise in healthy volunteers. Agreement was assessed using RR interval measurements, heart-rate metrics, and complementary agreement analyses, including participant-level correlation and Bland–Altman evaluation.

## Introduction

Cardiac rhythm monitoring is a cornerstone of cardiovascular diagnostics, enabling the detection of arrhythmias and the correlation of symptoms with electrocardiographic findings.^1^ While conventional 12-lead electrocardiography and multi-lead exercise electrocardiogram (ECG) systems provide reliable rhythm assessment in clinical settings, their use during dynamic physical activity remains challenging. The presence of multiple electrodes, wired connections, and external recording units may reduce comfort, impair signal stability, and limit feasibility during prolonged or high-intensity exercise.^2^

These limitations are particularly relevant in physically active individuals and athletes, in whom clinically relevant arrhythmias may occur preferentially during exercise or recovery phases.^2^ Reliable ECG monitoring under dynamic conditions is therefore important not only for exercise testing but also for sports cardiology, athlete surveillance, and physiological performance assessment.

In parallel, wearable ECG technologies have rapidly evolved, offering compact and wireless solutions for continuous cardiac monitoring. Compared with photoplethysmography-based devices, ECG-based wearables directly record cardiac electrical activity and provide more accurate RR interval estimation, particularly during exercise and at higher heart rates.^3^ However, despite their increasing adoption, evidence regarding the accuracy of wearable single-lead ECG systems during graded physical exertion remains relatively limited. Most available validation studies have focused on resting conditions, heart-rate monitoring, or consumer-grade devices, whereas fewer investigations have assessed signal quality and RR interval agreement during progressive exercise.^3^

The wearable ECG system evaluated in the present study consists of a lightweight chest-band device incorporating dry textile electrodes and a wireless acquisition module specifically designed for high-mobility environments. Unlike traditional exercise ECG systems, it does not require adhesive electrodes or wired connections, and it was developed to maintain stable signal acquisition during conditions characterized by substantial body movement.

The present study was designed to perform a simultaneous comparative evaluation of this wearable single-lead ECG chest band against a conventional multi-lead exercise ECG system during both rest and graded exercise. By focusing on time-aligned RR interval measurements, heart-rate assessment, and rhythm monitoring, we aimed to determine whether this wearable system can provide reliable ECG-derived physiological measurements under dynamic exercise conditions.

## Methods

### Study design

This prospective observational pilot study was designed to evaluate the agreement between a wearable single-lead ECG chest band and a conventional multi-lead exercise ECG system during rest and graded exercise testing. All participants underwent simultaneous ECG acquisition with both systems under standardized resting conditions and during symptom-limited cycle ergometer exercise. The primary objective was to assess agreement in RR interval measurements between the two systems, while secondary objectives included comparison of heart-rate metrics and evaluation of technical feasibility during exercise.

### Study population

Twenty-one healthy male volunteers aged 18–35 years were prospectively enrolled. The study was conceived as a pilot validation investigation aimed at assessing the technical feasibility and agreement of a wearable ECG system during rest and exercise. Demographic and clinical characteristics of the study population are reported in *Table 1*.

**Table 1:** Demographic and clinical characteristics of the study population

Characteristics	Value
Mean age	31 ± 4 y
Mean height	178.8 ± 11 cm
Mean weight	80.7 ± 5 kg
Basal heart rate	81 ± 6 bpm
Basal systolic blood pressure	120 ± 10 mmHg
Basal diastolic blood pressure	67 ± 11 mmHg
Peak heart rate	180 ± 25 bpm
Peak systolic blood pressure	185 ± 15 mmHg
Peak diastolic blood pressure	85 ± 10 mmHg
Peak workload	225 ± 25

All participants were physically active and able to perform symptom-limited cycle ergometer exercise testing. Inclusion criteria were the absence of known cardiovascular disease, previous arrhythmias, or structural heart abnormalities. Exclusion criteria included any medical condition potentially affecting heart rate or ECG interpretation, use of medications influencing cardiac rhythm, dermatological intolerance to device materials, or refusal to provide informed consent.

Three participants were excluded from the final analysis because simultaneous ECG recordings were incomplete or did not allow reliable RR interval extraction and comparison between the two systems. Consequently, 18 participants were included in the final analysis.

All participants provided written informed consent prior to enrolment, and the study was conducted in accordance with the ethical principles of the Declaration of Helsinki.

### ECG monitoring systems

#### Wearable ECG chest band

**Figure 1:**
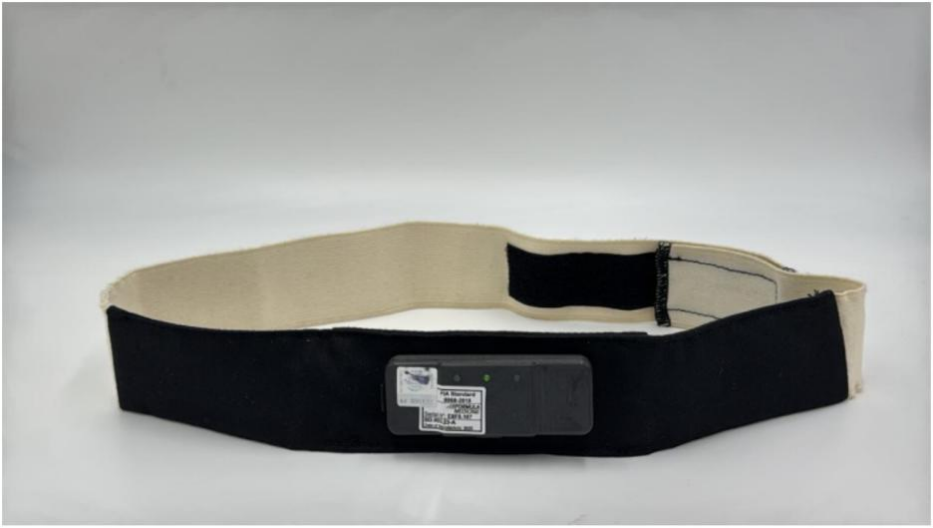
Biotronix ECG. It is flexible chest-band ECG monitoring system integrating two dry fabric electrodes embedded within an elastic thoracic strap. Weight: 25 g, dimensions: 77 × 28 × 14 mm.

The investigational device (Biotronix ECG; Litespeed AG, Switzerland), distributed by Formula Medicine SRL and certified according to FIA Standard 8868-2018 for biometric monitoring applications, is a wearable chest-band ECG monitoring system incorporating two dry textile electrodes embedded within an elastic thoracic strap. The device acquires a modified single-lead ECG signal configuration broadly comparable to Lead I (*Figure 1*).

The system was designed for continuous ECG acquisition during physical activity without the need for adhesive electrodes. ECG signals are recorded at a sampling frequency of 500 Hz and transmitted wirelessly via Bluetooth low energy for real-time visualization and storage.

The elastic chest-band configuration is intended to maintain electrode–skin contact throughout exercise and to reduce motion-related artefacts commonly encountered during dynamic movements. Unlike conventional exercise ECG systems, the device does not require wired connections or multiple adhesive electrodes, potentially improving comfort and practicality during prolonged or high-intensity physical activity.

Simultaneous ECG recordings from the wearable device and the reference multi-lead exercise ECG system were obtained in all study participants.

#### Reference ECG system

A conventional multi-lead exercise ECG system (Quark T12x, COSMED, Rome, Italy) was used as the reference method. The system provides continuous multi-lead ECG acquisition during exercise testing and is routinely employed for clinical exercise stress testing and physiological monitoring.

Surface electrodes were positioned according to the manufacturer's recommendations. ECG recordings from the reference system were acquired simultaneously with those obtained from the wearable chest-band device throughout both the resting and exercise phases of the study protocol. The reference system sampled ECG signals at approximately 100 Hz.

#### Study protocol

The study protocol consisted of two consecutive phases.

##### Resting phase

Simultaneous ECG recordings were obtained from both the wearable chest-band device and the reference multi-lead exercise ECG system while participants were resting in the supine position. Resting recordings were acquired for 30 s under stable conditions before the start of exercise.

##### Exercise phase

All participants underwent symptom-limited graded cycle ergometer testing using a standardized incremental workload protocol. Workload was progressively increased at predefined intervals until volitional exhaustion or achievement of standard test termination criteria. Simultaneous ECG recordings from both systems were acquired continuously throughout the entire exercise test.

For comparative analysis, the overlapping period of simultaneous acquisition was identified and used for RR interval extraction and comparison between devices. In addition, representative time-matched ECG segments from each workload stage were visually reviewed to assess signal quality and ECG interpretability across increasing levels of physiological stress and heart-rate response.

### Data collection

Continuous ECG recordings were acquired simultaneously using the wearable chest-band device and the reference multi-lead exercise ECG system during both rest and graded exercise testing.

The primary endpoint of the study was agreement in RR interval measurements between the two systems. Secondary assessments included comparison of heart-rate metrics, technical feasibility, signal quality, and rhythm monitoring during exercise.

Signal quality was evaluated by visual inspection of ECG recordings. Noise was defined as any ECG segment that could not be reliably interpreted because of motion artefacts, electrical interference, signal loss, or inadequate electrode contact. ECG recordings were considered suitable for analysis when QRS complexes could be clearly identified and RR intervals reliably extracted throughout the overlapping recording period.

All recordings were reviewed for the presence of cardiac arrhythmias. Premature atrial contractions, premature ventricular contractions, and atrial fibrillation were identified by visual analysis. Atrial and ventricular couplets were defined as two consecutive premature beats, whereas runs of supraventricular or ventricular tachycardia were defined as three or more consecutive premature beats. Given the low arrhythmic burden expected in this healthy volunteer cohort, arrhythmia assessment was considered exploratory.

Because the wearable device acquires a modified single-lead ECG signal, whereas the reference system provides conventional multi-lead recordings, direct comparison of ECG waveform morphology was not performed. Accordingly, the analysis focused on quantitative ECG-derived measurements, including RR intervals and heart-rate assessment, as well as qualitative evaluation of rhythm concordance between systems.

#### Signal processing and ECG comparison

ECG signals recorded by the wearable chest-band device and the reference multi-lead exercise ECG system were exported as raw digital time series and processed offline using a custom signal-processing pipeline implemented in R (version 4.4.3) with integrated Python modules.

To ensure temporal comparability between recordings, pre-processing steps were applied to account for differences in sampling frequency. The wearable ECG signal, originally acquired at 500 Hz, was down-sampled to 100 Hz using a fourth-order Butterworth low-pass filter (cut-off frequency 40 Hz) to prevent aliasing, followed by uniform resampling. The reference ECG system acquired signals at approximately 100 Hz and therefore required no additional resampling. Device timestamps were used to identify the overlapping period of simultaneous acquisition and to align the two recordings.

Subsequent signal processing was applied identically to both signals. A band-pass filter (3–40 Hz) was used to reduce baseline drift and high-frequency noise, followed by detrending to remove slow amplitude variations related to motion or respiration. R-wave detection was performed using the NeuroKit2 algorithm, which combines adaptive thresholding and waveform morphology criteria. Detected R peaks were converted into time stamps, and RR intervals were calculated as the time difference between consecutive beats.

For comparison of RR interval dynamics throughout the entire recording period, RR interval series from both devices were interpolated onto a common regular time grid at 100 Hz using cubic spline interpolation. This procedure was performed exclusively to achieve temporal alignment between recordings and did not alter the original RR interval measurements. Because interpolated samples do not represent independent physiological observations, agreement metrics derived from these series were calculated separately for each participant and subsequently summarized at the cohort level.

Agreement between the two systems was evaluated using complementary metrics, including Pearson correlation coefficient, mean absolute error (MAE), root mean square error (RMSE), and mean bias. In addition, agreement at the level of individual RR measurements was assessed using Bland–Altman analysis performed on directly paired beat-to-beat RR intervals derived from detected R peaks.

Recordings with insufficient signal quality or incomplete overlapping acquisition preventing reliable RR interval extraction were considered unsuitable for analysis.

### Statistical analysis

Continuous variables are presented as mean ± standard deviation when normally distributed, or as median and interquartile range otherwise. Normality was assessed using the Shapiro–Wilk test. Comparisons between paired measurements obtained from the two devices were performed using the paired Student's *t*-test or the Wilcoxon signed-rank test, as appropriate.

Pearson correlation coefficient, MAE, RMSE, and mean bias were calculated separately for each participant from the temporally aligned RR interval series and subsequently summarized at the cohort level. Bland–Altman analysis was performed using directly paired beat-to-beat RR intervals to estimate systematic bias and limits of agreement between devices.

Interpolation was used solely to achieve temporal alignment between recordings and was not intended to increase the number of independent physiological observations. Therefore, participant-level agreement metrics were considered the primary measures of device performance, and cohort-level results were derived from participant-level summaries rather than from the total number of interpolated samples.

A two-sided *P* value <0.05 was considered statistically significant. All statistical analyses and signal processing were performed using R software (version 4.4.3) with integrated Python-based signal-processing libraries.

### Study population

Eighteen healthy male volunteers (mean age 31 ± 4 years) completed the study protocol and were included in the final analysis. All participants successfully underwent simultaneous ECG acquisition during both rest and symptom-limited graded cycle ergometer exercise testing, achieving workloads of up to 275 W. No adverse events occurred during the study, and no participant required premature termination of the exercise protocol. Demographic and baseline characteristics of the study population are summarized in *Table 1*.

### Signal quality and feasibility

Simultaneous ECG acquisition using both the wearable chest-band device and the reference multi-lead exercise ECG system was successfully achieved in all participants during rest and graded exercise testing. ECG recordings obtained from both systems were generally of sufficient quality to allow RR interval extraction and comparative analysis throughout the study protocol.

Signal quality remained acceptable across the range of exercise workloads evaluated, and no systematic deterioration in ECG recordings was observed with increasing physical effort. Motion-related artefacts were occasionally present but did not prevent RR interval extraction in the recordings included in the final analysis. No device-related recording interruptions or Bluetooth transmission failures were observed.

Overall, the wearable ECG system proved technically feasible for continuous ECG acquisition during graded exercise testing and allowed reliable extraction of RR interval measurements under dynamic physiological conditions.

### Agreement in RR interval and heart rate measurements

RR interval series obtained from the wearable ECG device and the reference multi-lead ECG system demonstrated good overall agreement.

To characterize similarity between recordings over the entire duration of the exercise protocol, RR interval series were temporally aligned using interpolation onto a common 100-Hz time grid. This procedure generated 1 864 173 aligned samples and was used exclusively for temporal synchronization; these samples do not represent independent physiological observations.

**Figure 2:**
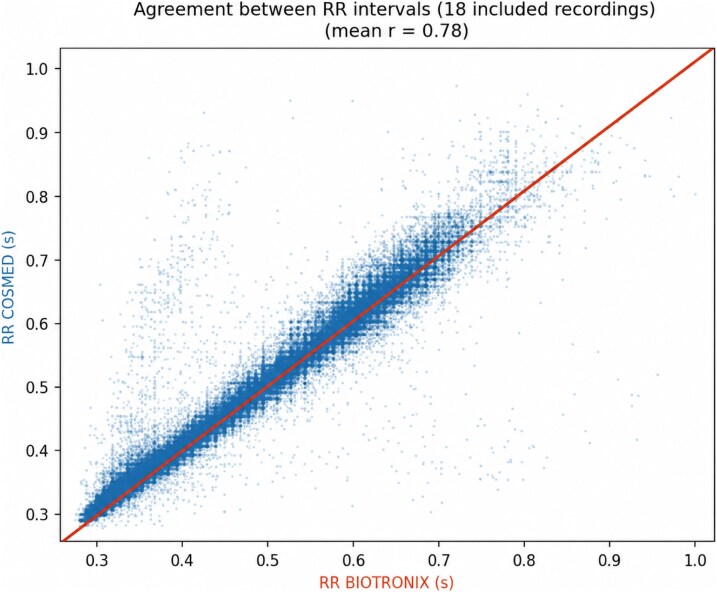
Agreement between RR intervals measured by the wearable chest-band ECG system (BIOTRONIX) and the reference multi-lead ECG system (COSMED). Scatter plot of temporally aligned RR interval samples obtained from the 18 participants included in the final analysis. RR interval series were interpolated and aligned onto a common 100-Hz time grid to enable direct temporal comparison between recordings. The red diagonal line represents the line of identity (y = x). The mean participant-level Pearson correlation coefficient was 0.78, demonstrating good agreement between the two systems across the range of physiological heart rates achieved during graded exercise testing.

**Figure 3:**
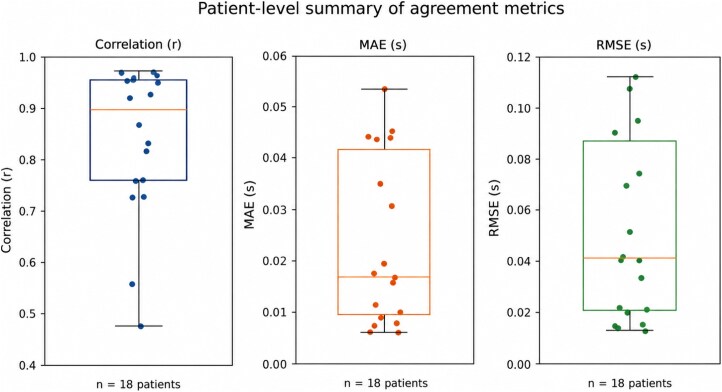
Distribution of participant-level agreement metrics. Box plots showing the distribution of Pearson correlation coefficient (*r*), MAE, and RMSE across the 18 participants included in the final analysis. Metrics were calculated from temporally aligned RR interval series obtained from the wearable chest-band ECG system (BIOTRONIX) and the reference multi-lead ECG system (COSMED). Individual participant values are displayed as superimposed points.

Participant-level agreement metrics derived from the aligned RR series showed good overall concordance between devices. Mean correlation was 0.77 ± 0.27 (median 0.88). MAE was 26 ms, mean bias was 13 ms, and RMSE was 66 ms. Scatter plots and participant-level analyses demonstrated consistent agreement across the range of RR intervals evaluated (*Tables 2* and *3, Figures 2* and *3*).

**Table 2:** Comparison of ECG-derived heart rate measurements between COSMED and BIOTRONIX (*n* = 18)

Variable	COSMED	BIOTRONIX	Mean difference (COSMED − BIOTRONIX)	*P*-value
Detected beats (*n*)	2152 ± 431	2206 ± 471	−54	0.028
Mean HR (bpm)	129.7 ± 12.7	132.4 ± 11.5	−2.63	0.009
Minimum HR (bpm)	62.0 ± 20.3	71.2 ± 16.7	−9.26	0.116
Maximum HR (bpm)	180.1 ± 11.8	175.9 ± 12.9	+4.21	0.099

Values are presented as mean ± standard deviation. Mean differences were calculated as COSMED minus BIOTRONIX.

**Table 3:** Agreement metrics derived from temporally aligned RR interval series (*n* = 18)

Metric	Value
Total temporally aligned RR samples	1 864 173
Pearson correlation (mean ± SD)	0.77 ± 0.27
Median correlation	0.88
MAE	26 ms
RMSE	66 ms
Mean bias	13 ms
Standard deviation of differences	60 ms
Limits of agreement	−0.11 to 0.14 s

RR interval series were temporally aligned by cubic spline interpolation onto a common 100-Hz time grid. Metrics are reported at the participant level and summarized across the study population.

**Figure 4:**
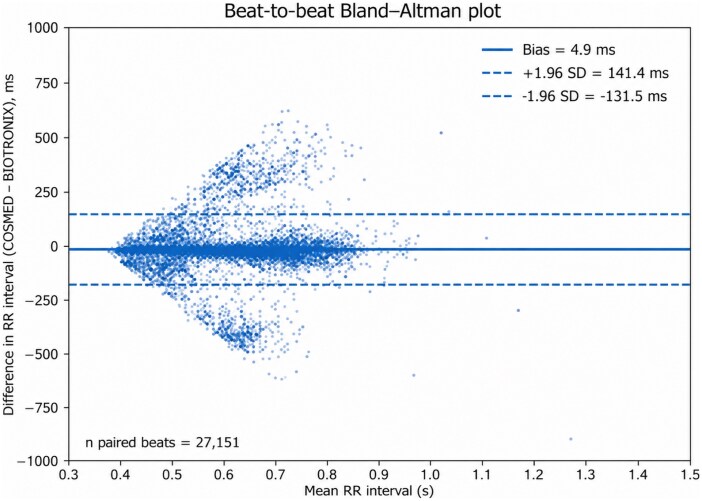
Beat-to-beat Bland–Altman analysis of RR interval agreement between the wearable chest-band ECG system (BIOTRONIX) and the reference multi-lead ECG system (COSMED). Bland–Altman plot showing agreement between directly paired beat-to-beat RR intervals obtained from the 18 participants included in the final analysis. The x-axis represents the mean RR interval measured by the two systems, while the y-axis represents the difference between measurements (COSMED − BIOTRONIX). A total of 27 151 paired RR intervals were analysed. The solid horizontal line indicates the mean bias (4.9 ms), while the dashed lines represent the 95% limits of agreement (−131.5 ms to +141.4 ms). The small overall bias and relatively narrow limits of agreement support good beat-to-beat agreement between the wearable ECG system and the reference multi-lead ECG system across a broad range of physiological RR intervals.

Agreement at the level of individual RR measurements was assessed using directly paired beat-to-beat RR intervals derived from detected R peaks. A total of 27 151 paired RR intervals were available for comparison. Bland–Altman analysis demonstrated a mean bias of 4.9 ms, with 95% limits of agreement ranging from −131.5 ms to +141.4 ms (*Figure 4*).

Mean heart-rate values were strongly correlated between devices (*r* = 0.96), with a small but statistically significant difference (COSMED − BIOTRONIX: −2.6 bpm, *P* = 0.009). In contrast, minimum and maximum heart-rate values showed weaker agreement and no statistically significant differences (*P* > 0.05). The reference system identified slightly more beats than the wearable device, although agreement remained excellent (*r* = 0.98).

Overall, participant-level agreement metrics, beat-to-beat analysis, and heart-rate comparisons consistently demonstrated good agreement between the wearable ECG device and the reference multi-lead ECG system.

### Rhythm classification and arrhythmia detection

No sustained supraventricular or ventricular arrhythmias were observed during the study protocol. Occasional premature atrial and ventricular beats were identified in a small number of participants and were similarly recognized by both monitoring systems. Given the low arrhythmic burden of this healthy volunteer cohort, no formal analysis of arrhythmia detection performance was performed.

## Discussion

In this pilot validation study, a wearable single-lead ECG chest band demonstrated good agreement with a conventional multi-lead exercise ECG system during both rest and graded exercise testing. In the final cohort of 18 participants, participant-level analysis of temporally aligned RR interval series showed a MAE of 26 ms, a mean bias of 13 ms, and an RMSE of 66 ms, with a mean correlation coefficient of 0.77 ± 0.27 (median 0.88). Importantly, agreement remained consistent when assessed using directly paired beat-to-beat RR intervals, supporting the robustness of the findings independently of the interpolation procedure used for temporal alignment. Taken together, these results indicate that the wearable system provides reliable RR interval measurements across a broad range of physiological heart rates achieved during graded exercise testing.

### Principal findings

The principal finding of the present study is the ability of the wearable ECG chest band to provide reliable RR interval measurements during both rest and exercise. While wearable ECG systems are frequently validated under resting conditions, their performance during dynamic exercise remains challenging because of motion artefacts, sweating, and changes in electrode–skin contact.^2,3^

In the present study, simultaneous ECG acquisition was successfully achieved throughout the exercise protocol, and signal quality was generally adequate to allow RR interval extraction and comparison between systems. The primary analysis, based on temporally aligned RR interval series, included more than 1.8 million interpolated samples and demonstrated good agreement between the wearable device and the reference multi-lead ECG system. Importantly, a secondary beat-to-beat analysis yielded consistent results, confirming that the observed agreement was not dependent on the interpolation procedure used for temporal alignment.

Agreement was stronger when assessed on RR interval measurements than on derived heart-rate metrics. Mean heart-rate values were highly correlated between systems (*r* = 0.96), although a small systematic difference was observed (−2.6 bpm). These findings support the use of RR interval–based metrics as the primary endpoint for validation studies of wearable ECG systems.^1^

### Clinical relevance and relationship with existing wearable technologies

Monitoring cardiac rhythm during physical activity remains challenging with conventional multi-lead systems because of electrode instability, motion artefacts, and limited user comfort. This is particularly relevant in athletes and physically active individuals, in whom clinically relevant arrhythmias may occur preferentially during exercise or recovery phases.^2^ The ability to acquire reliable ECG-derived RR intervals under physiological stress conditions expands the potential of wearable monitoring beyond resting evaluations and controlled clinical environments.^5–7^

Wearable technologies for cardiac monitoring have expanded rapidly in recent years.^3^ While many commercially available devices rely on photoplethysmographic sensors, ECG-based wearables provide direct assessment of cardiac electrical activity and RR intervals.^1,3,4^ Smartwatches, adhesive ECG patches, and chest-band systems each offer specific advantages and limitations with respect to signal quality, duration of monitoring, and usability during exercise. The present findings support the feasibility of a chest-band ECG configuration during graded exercise, demonstrating good agreement with a conventional multi-lead ECG system across a broad range of physiological heart rates. These characteristics may make chest-band systems particularly attractive for continuous rhythm monitoring in physically active individuals.

Although the present study was performed in healthy young adults, the demonstrated agreement in RR interval measurements supports the potential application of this technology in broader populations, including older individuals and patients at increased arrhythmic risk. Future studies should evaluate its diagnostic performance in clinical cohorts with documented arrhythmias or structural heart disease.

### Implications for high-performance environments

The ability of the wearable chest-band ECG system to provide reliable RR interval measurements during exercise suggests potential applications beyond conventional clinical and sports settings. In particular, environments characterized by extreme physiological stress—such as high-speed motorsport—require monitoring systems that combine minimal invasiveness, user comfort, and robustness to motion-related artefacts.

Although the present study was not conducted in motorsport environments, the observed agreement with a conventional multi-lead ECG system provides preliminary support for further investigations under extreme real-world conditions. Future studies should specifically evaluate device performance during high-performance racing and other settings characterized by intense physical and environmental stress.^8^

### Future perspectives

Beyond exercise ECG monitoring, wearable chest-band ECG systems may contribute to the growing field of digital cardiology. Continuous ECG acquisition combined with cloud-based analytics and artificial intelligence may facilitate longitudinal rhythm surveillance, remote monitoring, and personalized cardiovascular assessment. Future studies should evaluate these technologies in larger and more diverse populations, including athletes, patients with known arrhythmias, and individuals undergoing cardiovascular rehabilitation.^3^

## Limitations

This was a single-centre pilot study with a limited sample size of healthy young volunteers. The study was not powered to assess diagnostic accuracy for rare or complex arrhythmias, and the low arrhythmic burden limits conclusions regarding performance for clinically relevant rhythm disorders.

The wearable device records a single modified ECG lead, whereas the reference system provides conventional multi-lead recordings; therefore, ECG morphology analysis and arrhythmia localization were not assessed. In addition, the study population consisted exclusively of healthy male participants, limiting the generalizability of the findings to women, older individuals, and patients with cardiovascular disease.

Agreement analysis primarily relied on temporally aligned RR interval series obtained through interpolation to a common time grid. Although a complementary beat-to-beat sensitivity analysis yielded consistent results, interpolation-based approaches may not fully reflect all aspects of beat-level agreement.

Finally, exercise testing was performed in a controlled laboratory setting using cycle ergometer exercise and may not fully reproduce real-world training environments, competitive sports settings, or extreme physiological conditions such as those encountered in professional motorsport.

## Conclusions

In this pilot validation study, a wearable single-lead ECG chest band demonstrated good agreement with a conventional multi-lead exercise ECG system for RR interval measurement during both rest and graded exercise testing. Participant-level agreement was high, with low measurement error and limited systematic bias across a broad range of physiological heart rates. Consistent findings obtained from both temporally aligned RR series and direct beat-to-beat analyses support the robustness of the observed agreement.

Although not intended to replace diagnostic multi-lead ECG systems, this wearable device provides reliable RR interval–based monitoring and may serve as a practical complementary tool for continuous cardiac rhythm surveillance in physically active individuals.

These findings support further investigation of wearable ECG systems in larger and more diverse populations, including patients with cardiovascular disease, as well as in high-performance and extreme environments where robust, unobtrusive, and continuous physiological monitoring is required.

## Data Availability

The data underlying this article are available from the corresponding author upon reasonable request.

## Lead author biography

Valentina Barletta, MD, PhD, is a cardiologist at the Azienda Ospedaliero-Universitaria Pisana, where she is involved in clinical practice focusing on cardiac arrhythmias and advanced cardiovascular imaging. She obtained her PhD in Clinical and Translational Sciences and has recently completed a Master’s degree in Cardiovascular Imaging, as well as an advanced training programme in cardiomyopathies across paediatric and adult populations. Her research interests include multimodality imaging applied to electrophysiology, atrial fibrillation, and cardiomyopathies. She also has several years of experience as a physician in motorsport, working closely with professional drivers and contributing to cardiovascular assessment and monitoring in high-performance settings.
